# Supplementary material for: Branched-chain amino acid aminotransferase 2 regulates ferroptotic cell death in cancer cells
Source: Cell Death Differ. 2020 Oct 23;28(4):1222–36. doi: 10.1038/s41418-020-00644-4 (PMC8027606; doi:10.1038/s41418-020-00644-4)
Supplement: Supplementary file 1 — Supplementary Information [file 41418_2020_644_MOESM1_ESM.docx]

Supplementary Information for:

**Branched Chain Amino Acid Aminotransferase 2 Regulates Ferroptotic Cell Death in Cancer Cells**

Kang Wang^1#^, Zhengyang Zhang^1#^, Hsiang-i Tsai^1#^, Yanfang Liu^2^, Jie Gao^1^, Ming Wang^1^, Lian Song^1^, Xiongfeng Cao^1^, Zhanxue Xu^3^, Hongbo Chen^3^, Aihua Gong^2^, Dongqing Wang^1*^, Fang Cheng^1,3,4*^, Haitao Zhu^1*^

**Figure legends**

**Figure S1.** (A) Aspc-1, HepG2, SW480 and HT1080 cells were treated with DMSO (control), erastin (10 or 20 μmol/L), sorafenib (5 or 10 μmol/L) or sulfasalazine (1 or 2 mmol/L) in the absence or presence of ferrostatin-1(1 μmol/L), ZVAD-FMK (1 μmol/L), and Necrosulfonamide (0.5 μmol/L) for 48 h, and cell viability was assayed by CCK8. (B) Aspc-1 and HepG2 cells treated with DMSO (control), erastin (10 or 20 μmol/L), sorafenib (5 or 10 μmol/L) or sulfasalazine (1 or 2 mmol/L) in the absence or presence of ferrostatin-1(1 μmol/L). Images show live control cells and dead cells (Sytox Green+). SOR represents for sorafenib; SAS represents for sulfasalazine; Fer-1 represents for ferrostatin-1. Experiments were repeated three times, and the data are expressed as the mean ± SEM. **p* < 0.05 vs. control group. Statistical analysis was performed using Student's t-test.

**Figure S2.** (A) qRT-PCR analysis of the mRNA expression level of BCAT2 and BCAT1 in Aspc-1, HepG2, SW480 and HT1080 cells treated with erastin (10 or 20 μmol/L), sorafenib (5 or 10 μmol/L) or sulfasalazine (1 or 2 mmol/L). GAPDH was detected as a loading control. (B) Quantitative analysis of the protein expression levels of BCAT2 and BCAT1 in Aspc-1, HepG2, SW480 and HT1080 cells treated with erastin (10 or 20 μmol/L), sorafenib (5 or 10 μmol/L) or sulfasalazine (1 or 2 mmol/L). β-tubulin expression was detected as a loading control. (C) Western blot analysis of the protein expression levels of BCAT2 in HepG2 cells treated with erastin (20 μmol/L), sorafenib(10 μmol/L) or sulfasalazine(2 mmol/L) for the indicated time(0, 4, 8, 12, 24, 48, 72 h). β-tubulin expression was detected as a loading control. SOR represents for sorafenib; SAS represents for sulfasalazine. Experiments were repeated three times, and the data are expressed as the mean ± SEM. **p* < 0.05 vs. control group. Statistical analysis was performed using Student's t-test.

**Figure S3. The clinical significance BCAT2 in hepatocellular carcinoma patients** (A) Analysis of the TCGA database for the correlation BCAT2 mRNA expression level with hepatocellular carcinoma TNM staging. (B) Analysis of the TCGA database for the correlation BCAT2 mRNA expression level with the other ferroptosis (GPX4, TP53, NRF2 and SLC7A11) and autophagy (NCOA4, BECN1) related markers. The results are presented by heat map: n =390.

**Figure S4.** (A, B) Quantitative analysis of the ratio of pAMPK(T172) to AMPK(A) and protein expression levels of SREBP1(B) in Aspc-1 and HepG2 cells treated with erastin (10 or 20 μmol/L), sorafenib (5 or 10 μmol/L) or sulfasalazine (1 or 2 mmol/L). β-tubulin expression was detected as a loading control. (C, D) Quantitative analysis of the ratio of pAMPK(T172) to AMPK(C) and protein expression levels of SREBP1(D) in Aspc-1 and HepG2 cells treated with erastin (10 or 20 μmol/L), sorafenib(5 or 10 μmol/L) or sulfasalazine(1 or 2 mmol/L) in the absence or presence of AICAR (AMPK activator, 2 mmol/L) and Compound C (AMPK inhibitor, 1 μmol/L). β-tubulin expression was detected as a loading control. AMPK represents for AMP-activated protein kinase; SREBP1 represents for sterol response element binding protein 1; pAMPK-T172 represents for AMPK phosphorylation on threonine residue 172 (T172); CC represents for Compound C; SOR represents for sorafenib; SAS represents for sulfasalazine. Experiments were repeated three times, and the data are expressed as the mean ± SEM. **p* < 0.05, vs. control group. Statistical analysis was performed using Student's t-test.

**Figure S5.** (A) Western blot analysis of SREBP1 expression in SREBP1-knockout HepG2 cells using CRISPR-Cas9 technology. (B) Western blot analyses of shRNA knockdown efficiency of SREBP1 protein in HepG2 cells. (C, D) Western blot analysis of the protein expression levels of BCAT2 and SREBP1 in SREBP1-knockout and parental HepG2 cells treated with or without erastin (20 μmol/L), sorafenib (10 μmol/L) or sulfasalazine (2 mmol/L). (E, F) Western blot analysis of the protein expression levels of BCAT2 and SREBP1 in SREBP1-knockdown and parental HepG2 cells treated with or without erastin (20 μmol/L), sorafenib (10 μmol/L) or sulfasalazine (2 mmol/L). β-tubulin expression was detected as a loading control. SOR represents for sorafenib; SAS represents for sulfasalazine. Experiments were repeated three times, and the data are expressed as the mean ± SEM. **p* < 0.05, vs. control group. Statistical analysis was performed using Student's t-test.

**Figure S6.** (A) Western blot analysis of the protein expression levels of NCOA4 in Aspc-1 and HepG2 cells treated with DMSO(control), erastin (10 or 20 μmol/L), sorafenib (5 or 10 μmol/L) or sulfasalazine (1 or 2 mmol/L). β-tubulin expression was detected as a loading control. (B) HepG2 cells were transfected with a GFP-LC3 plasmid for 24 h and then treated with erastin (20 μmol/L), sorafenib (10 μmol/L) or sulfasalazine (2 mmol/L) for 24 h. The GFP-LC3 puncta were assayed using image analysis. (C) Western blot analysis of NCOA4 expression in NCOA4-knockdown Aspc-1 and HepG2 cells. (D) The relative levels of Fe^2+^ were assayed in NCOA4-knockdown and parental Aspc-1 and HepG2 cells were treated with DMSO (control), erastin (10 or 20 μmol/L), sorafenib (5 or 10 μmol/L) or sulfasalazine (1 or 2 mmol/L). (E) Western blot analysis of ATG7 expression in ATG7-knockdown and parental Aspc-1 and HepG2 cells. (F) The relative levels of Fe^2+^ were assayed in ATG7-knockdown and parental Aspc-1 and HepG2 cells were treated with DMSO (control), erastin (10 or 20 μmol/L), sorafenib (5 or 10 μmol/L) or sulfasalazine (1 or 2 mmol/L). SOR represents for sorafenib; SAS represents for sulfasalazine. Experiments were repeated three times, and the data are expressed as the mean ± SEM. **p* < 0.05 vs. control group. Statistical analysis was performed using Student's t-test.

**Figure S7.** Western blot analysis of the protein expression levels of BCAT2, pAMPK-T172 and AMPK in Aspc-1 and HepG2 cells treated with erastin (10 or 20 μmol/L), sorafenib (5 or 10 μmol/L) or sulfasalazine (1 or 2 mmol/L) in the absence or presence of BafA1(20nM) or DFO (100 μmol/L). β-tubulin expression was detected as a loading control. SOR represents for sorafenib; SAS represents for sulfasalazine; DFO represents for deferoxamine mesylate. Experiments were repeated three times, and the data are expressed as the mean ± SEM. **p* < 0.05 vs. control group. Statistical analysis was performed using Student's t-test.

**Figure S8. BCAT2 is required for system Xc^–^ inhibitor induced ferroptosis.** (A) Western blot analysis of BCAT2 expression in BCAT2 overexpressed Aspc-1 and HepG2 cells. (B, C, D) BCAT2 overexpressed and parental Aspc-1 cells were treated with DMSO (control), erastin (10 μmol/L), sorafenib (5 μmol/L) or sulfasalazine (1 mmol/L). The relative levels of Fe^2+^ (B), ratio of GSH/GSSG (C), and MDA (D) were assayed. (E, F, G) BCAT2 overexpressed and parental Aspc-1 cells were treated with DMSO (control), erastin (10 μmol/L), sorafenib (5 μmol/L) or sulfasalazine (1 mmol/L) for the indicated time (0, 12, 24, 48 h). The relative levels of intracellular glutamate (E), glutamate release (F), and cell viability (G) were assayed. (H) C57BL/6 were injected subcutaneously with 1 × 10^6^ BCAT2 overexpressed and parental Panc02 cancer cells and treated with or without erastin (40 mg/kg/i.t., every two says) and/or NAC (administered in drinking water at 1 g/L) at day 7 for 2 weeks (n = 5 mice/group). Tumor volume was calculated every two days for 2 weeks. (I)Representative photographs of isolated tumor tissues at day 14 after treatment. MDA represents for malondialdehyde; GSH represents for glutathione; GSSG represents for oxidized glutathione; SOR represents for sorafenib; SAS represents for sulfasalazine; NAC represents for N-acetyl-cysteine; i.t. represents for intratumoral injection. Experiments were repeated three times, and the data are expressed as the mean ± SEM. **p* < 0.05 vs. control group. Statistical analysis was performed using Student's t-test.

**Figure S9.** (A, B, C) BCAT2 overexpressed and parental HepG2 cells were treated with DMSO (control), RSL3(5 μmol/L), and BSO (1 mmol/L). The relative levels of Fe^2+^ (A), MDA (B), and ratio of GSH/GSSG (C) were assayed. (D, E, F) BCAT2 overexpressed and parental HepG2 cells were treated with DMSO (control), RSL3 (5 μmol/L), and BSO (1 mmol/L) for the indicated time (0, 12, 24, 48 h). The relative levels of intracellular glutamate (D), glutamate release (E), and cell viability (F) were assayed. (G, H, I) BCAT2 overexpressed and parental Aspc1 cells were treated with DMSO (control), RSL3(5 μmol/L), and BSO (1 mmol/L). The relative levels of Fe^2+^ (G), ratio of GSH/GSSG (H), and MDA (I) were assayed. (J, K, L) BCAT2 overexpressed and parental Aspc-1 cells were treated with DMSO (control), RSL3(5 μmol/L), and BSO(1 mmol/L) for the indicated time(0, 12, 24, 48 h). The relative levels of intracellular glutamate (J), glutamate release (K), and cell viability (L) were assayed. MDA represents for malondialdehyde; GSH represents for glutathione; GSSG represents for oxidized glutathione; RSL3 represents for RAS-selective lethal 3; BSO represents for buthionine sulfoximine. Experiments were repeated three times, and the data are expressed as the mean ± SEM. **p* < 0.05 vs. control group. Statistical analysis was performed using Student's t-test.

**Figure S10.** (A) Western blot analysis the protein expression levels of BCAT2 and BCAT1 in HepG2 cancer cells treated with DMSO (control), RSL3 (5 μmol/L), BSO (1 mmol/L) or DON (25 μmol/L). β-tubulin expression was detected as a loading control. (B) Cell viability was assessed in BCAT2 overexpressed and parental HepG2 cells were treated with DMSO (control), erastin (10 μmol/L), sorafenib (5 μmol/L) or sulfasalazine (1 mmol/L) in the presence or absence of DON. RSL3 represents for RAS-selective lethal 3; BSO represents for buthionine sulfoximine; DON represents for 6-diazo-5-oxo-L-norleucine. Experiments were repeated three times, and the data are expressed as the mean ± SEM. **p* < 0.05 vs. control group. Statistical analysis was performed using Student's t-test.

**Figure S11.** (A) Western blot analysis of BCAT2 expression in BCAT2-knockdown Aspc-1 and HepG2 cells. (B) Mitochondria morphology of BCAT2 knockdown and parental HepG2 cells observed by TEM. White arrowhead indicates reduction of mitochondria crista. (C-H) BCAT2 knockdown and parental Aspc-1 cells were treated with DMSO (control), erastin (10 μmol/L), sorafenib (5 μmol/L) or sulfasalazine (1 mmol/L). The relative levels of Fe^2+^ (C), MDA (D), ratio of GSH/GSSG (E), intracellular glutamate (F), glutamate release (G) and cell viability (H) were assayed. (I) Colony-forming assay analysis of the colony formation ability of BCAT2-knockdown and parental HepG2 cells following 14 day culture. (J) Western blot analysis of the protein expression levels of BCAT2 in BCAT2-knockdown and parental Aspc-1 cells treated with or without erastin (10 μmol/L), sorafenib (5 μmol/L) or sulfasalazine (1 mmol/L). β-tubulin expression was detected as a loading control. MDA represents for malondialdehyde; GSH represents for glutathione; GSSG represents for oxidized glutathione; SOR represents for sorafenib; SAS represents for sulfasalazine. Experiments were repeated three times, and the data are expressed as the mean ± SEM. **p* < 0.05 vs. control group. Statistical analysis was performed using Student's t-test.

**Figure S12.** (A-F) BCAT2 knockdown and parental HepG2 cells were treated with DMSO (control), RSL3(5 μmol/L), or BSO(1 mmol/L). The relative levels of Fe^2+^ (A), ratio of GSH/GSSG(B), MDA (C), intracellular glutamate (D), glutamate release (E), cell viability (F) were assayed. (G-K) BCAT2 knockdown and parental Aspc1 cells were treated with DMSO (control), RSL3(5 μmol/L), or BSO(1 mmol/L). The relative levels of Fe^2+^ (G), ratio of GSH/GSSG(H), MDA (I), intracellular glutamate (J), glutamate release (K), cell viability (L) were assayed. RSL3 represents for RAS-selective lethal 3; BSO represents for buthionine sulfoximine. Experiments were repeated three times, and the data are expressed as the mean ± SEM. **p* < 0.05 vs. control group. Statistical analysis was performed using Student's t-test.

**Figure S13.** (A) Western blot analysis of the protein expression levels of SLC7A11 and GPX4 in BCAT2 knockdown and parental Aspc-1 and HepG2 cells. β-tubulin expression was detected as a loading control. (B) Western blot analysis of the protein expression levels of GPX4 in Aspc-1 and HepG2 cells treated with sorafenib (5 μmol/L), sulfasalazine (1 mmol/L) or sorafenib (5 μmol/L) + sulfasalazine (1 mmol/L). β-tubulin expression was detected as a loading control. SOR represents for sorafenib; SAS represents for sulfasalazine. Experiments were repeated three times, and the data are expressed as the mean ± SEM. **p* < 0.05 vs. control group. Statistical analysis was performed using Student's t-test.

**Figure S14.** (A)Western blot analysis of the protein expression level of BCAT2 in the isolated orthotopic hepatocellular carcinoma tissues tumor tissues at day 60 after treatment. (B)qRT-PCR analysis of mRNA expression level of BCAT2 in the isolated orthotopic hepatocellular carcinoma tissues tumor tissues at day 60 after treatment. (C) GSH levels in isolated tumors at day 60 after treatment were assayed. (D) MDA levels in isolated tumors at day 60 after treatment were assayed. MDA represents for malondialdehyde; GSH represents for glutathione; SOR represents for sorafenib; SAS represents for sulfasalazine. Experiments were repeated three times, and the data are expressed as the mean ± SEM. **p* < 0.05, ** *p* < 0.01, *** *p* < 0.001 vs. control group. Statistical analysis was performed using Student's t-test.
