# Supplementary figures and images for: Branched-chain amino acid aminotransferase 2 regulates ferroptotic cell death in cancer cells
Source: Cell Death Differ. 2020 Oct 23;28(4):1222–36. doi: 10.1038/s41418-020-00644-4 (PMC8027606; doi:10.1038/s41418-020-00644-4)

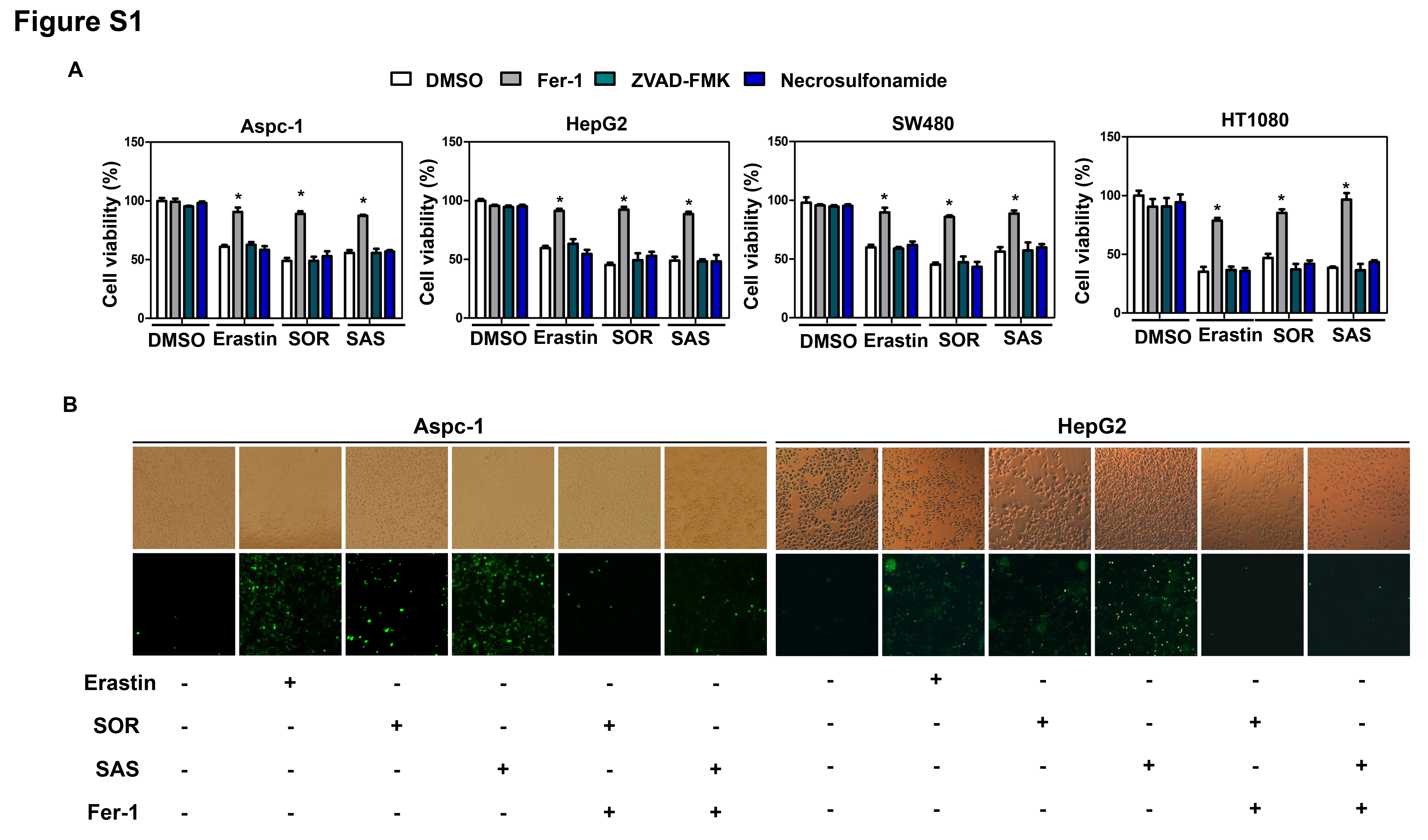

Supplement: Supplementary file 2 — Figure S1 [file 41418_2020_644_MOESM2_ESM.tif]

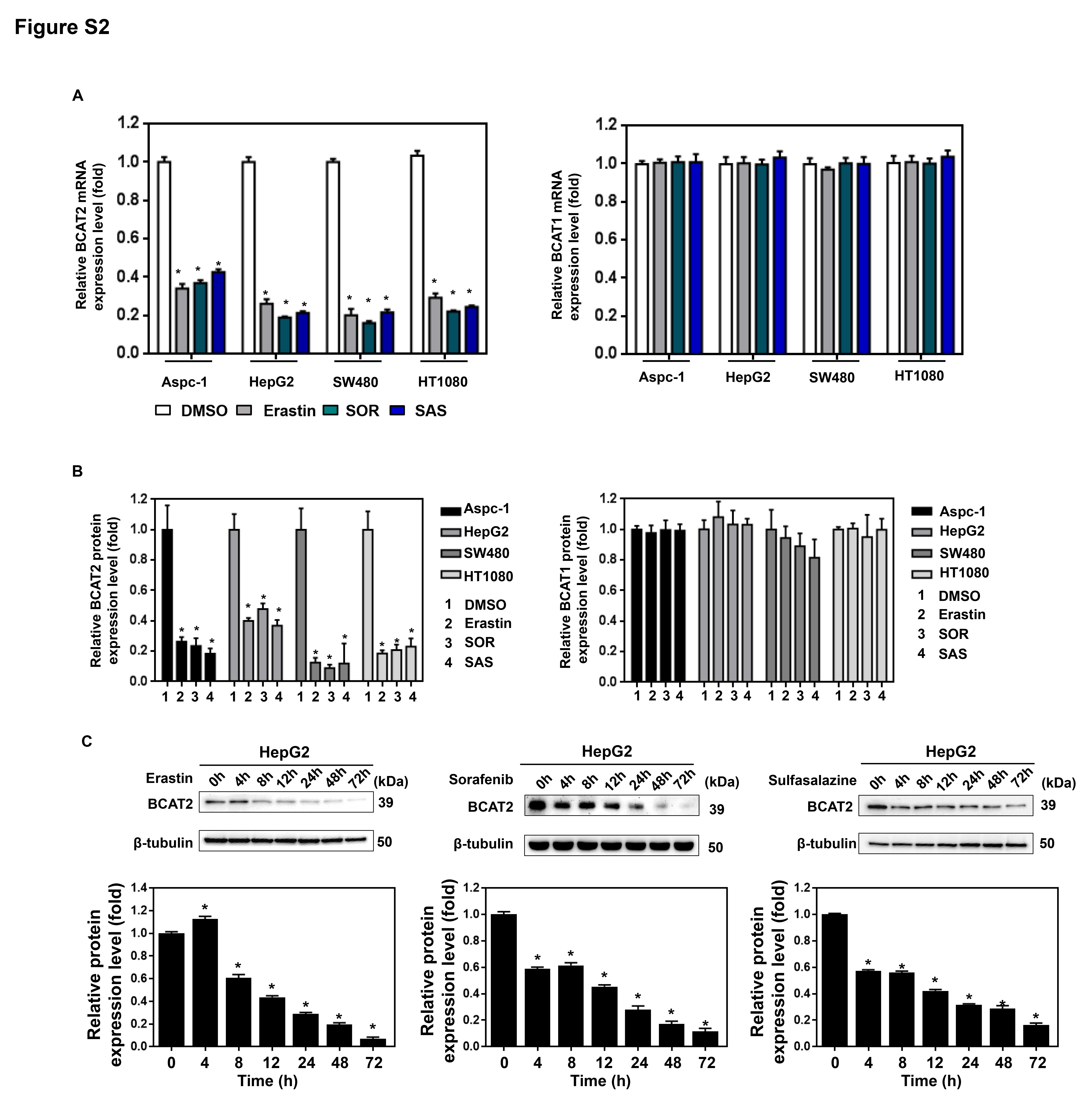

Supplement: Supplementary file 3 — Figure S2 [file 41418_2020_644_MOESM3_ESM.tif]

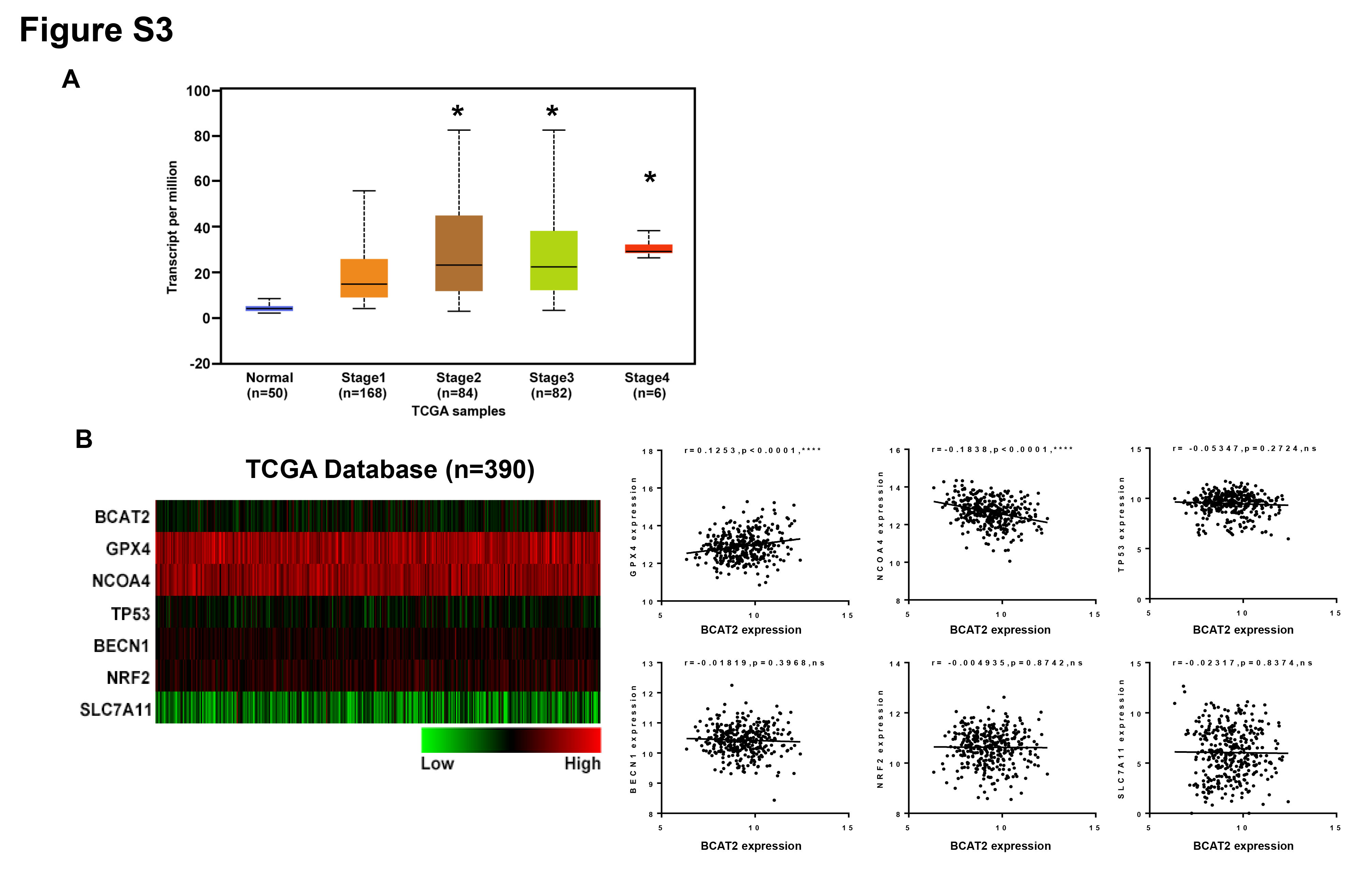

Supplement: Supplementary file 4 — Figure S3 [file 41418_2020_644_MOESM4_ESM.tif]

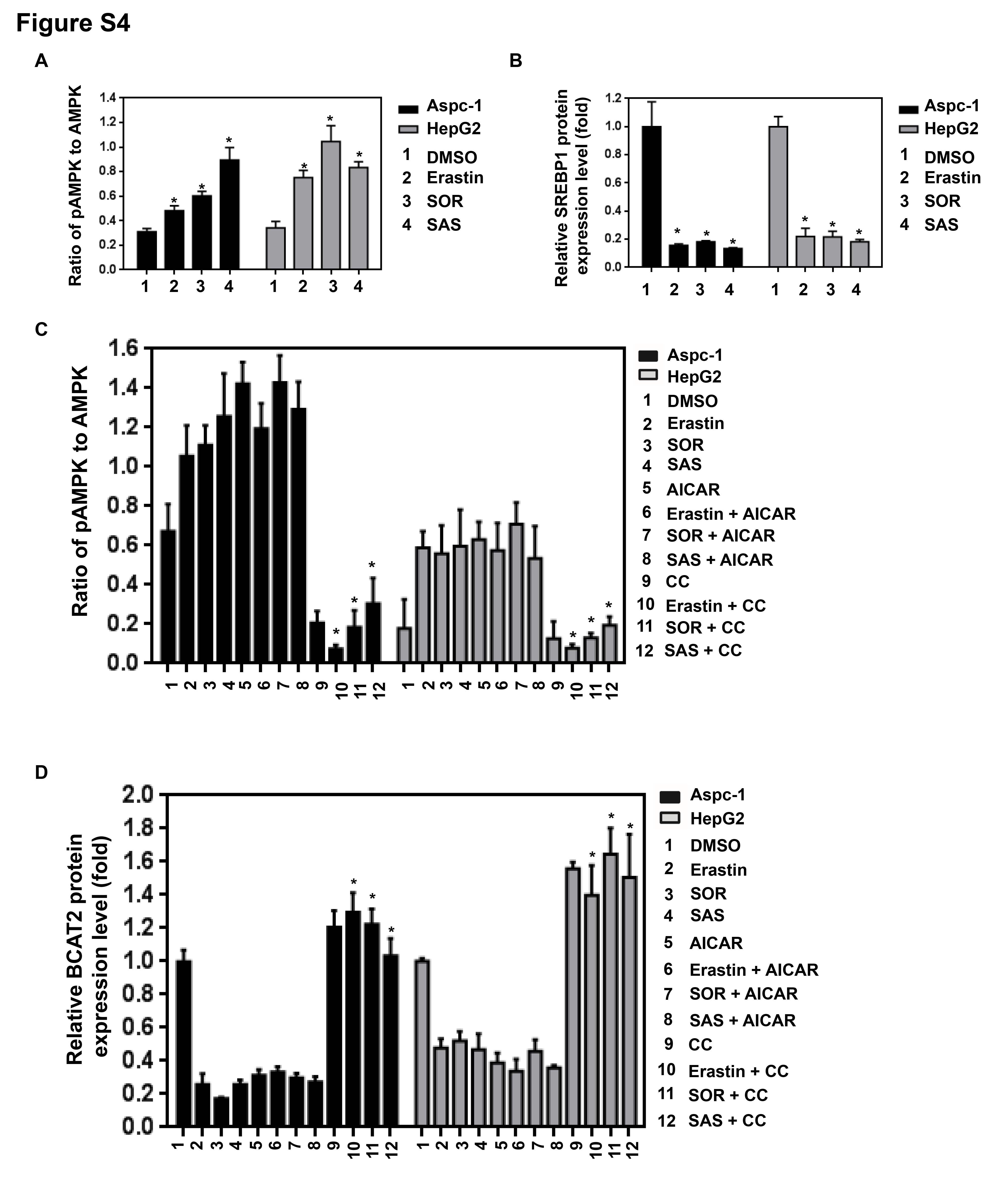

Supplement: Supplementary file 5 — Figure S4 [file 41418_2020_644_MOESM5_ESM.tif]

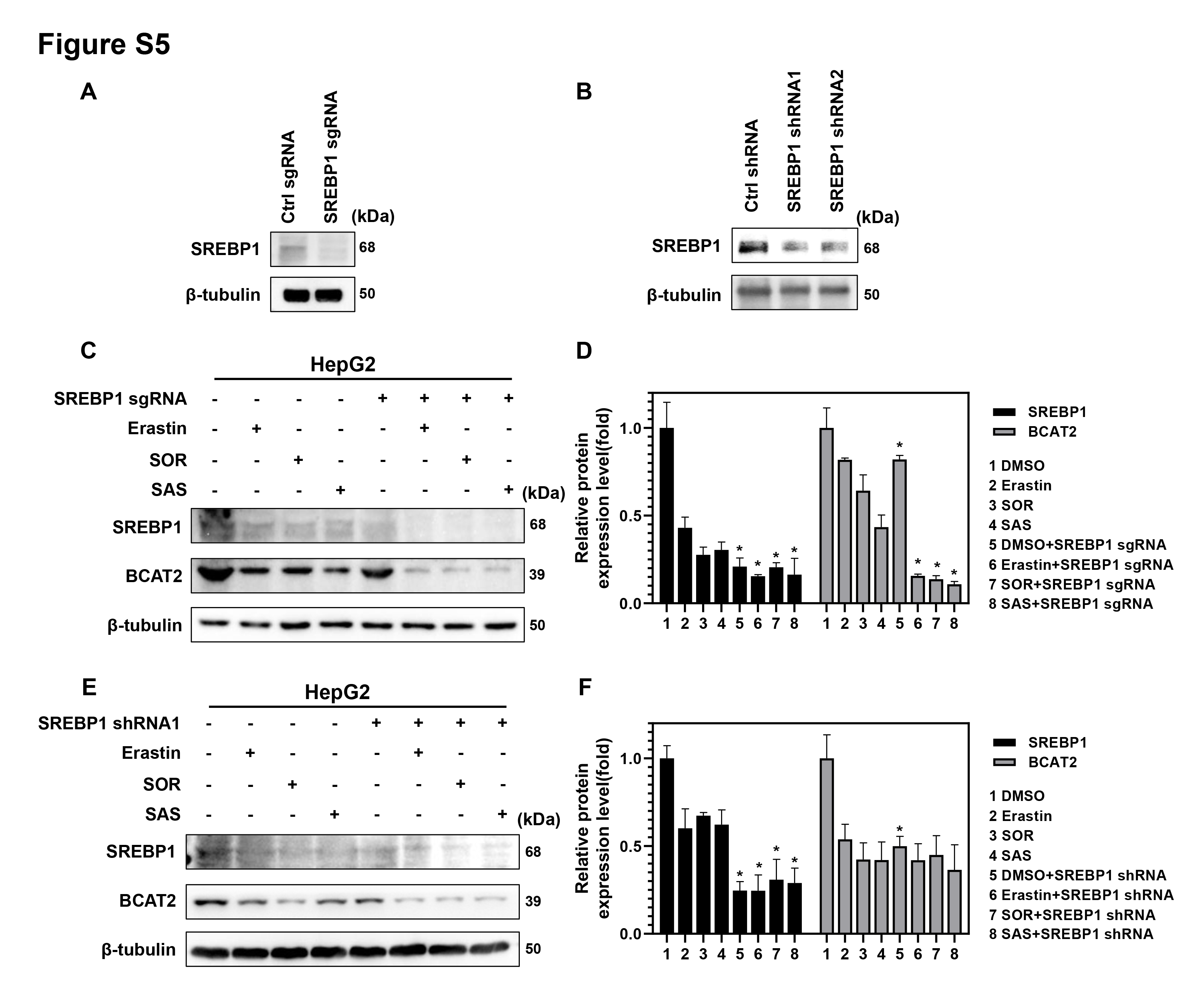

Supplement: Supplementary file 6 — Figure S5 [file 41418_2020_644_MOESM6_ESM.tif]

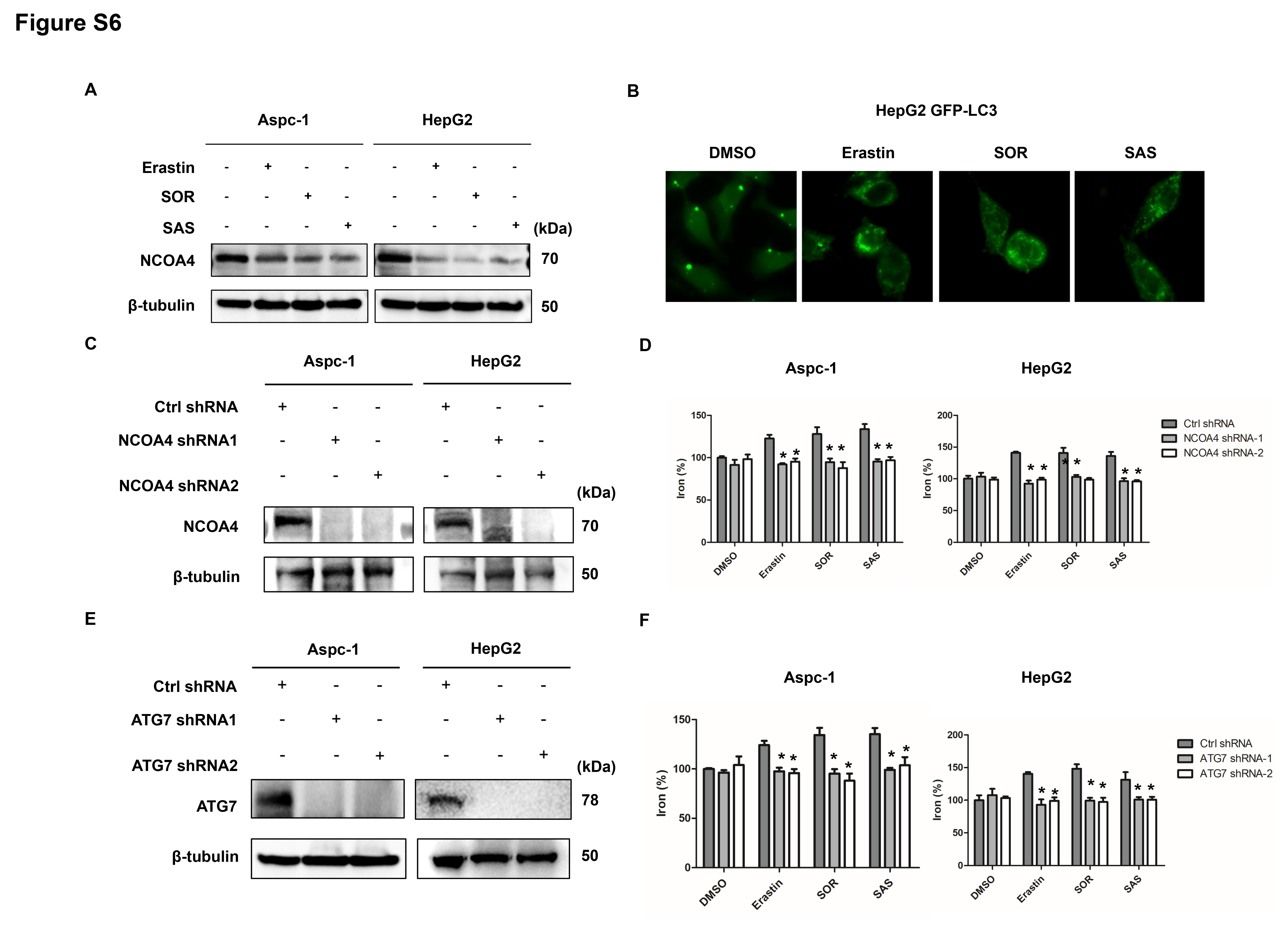

Supplement: Supplementary file 7 — Figure S6 [file 41418_2020_644_MOESM7_ESM.tif]

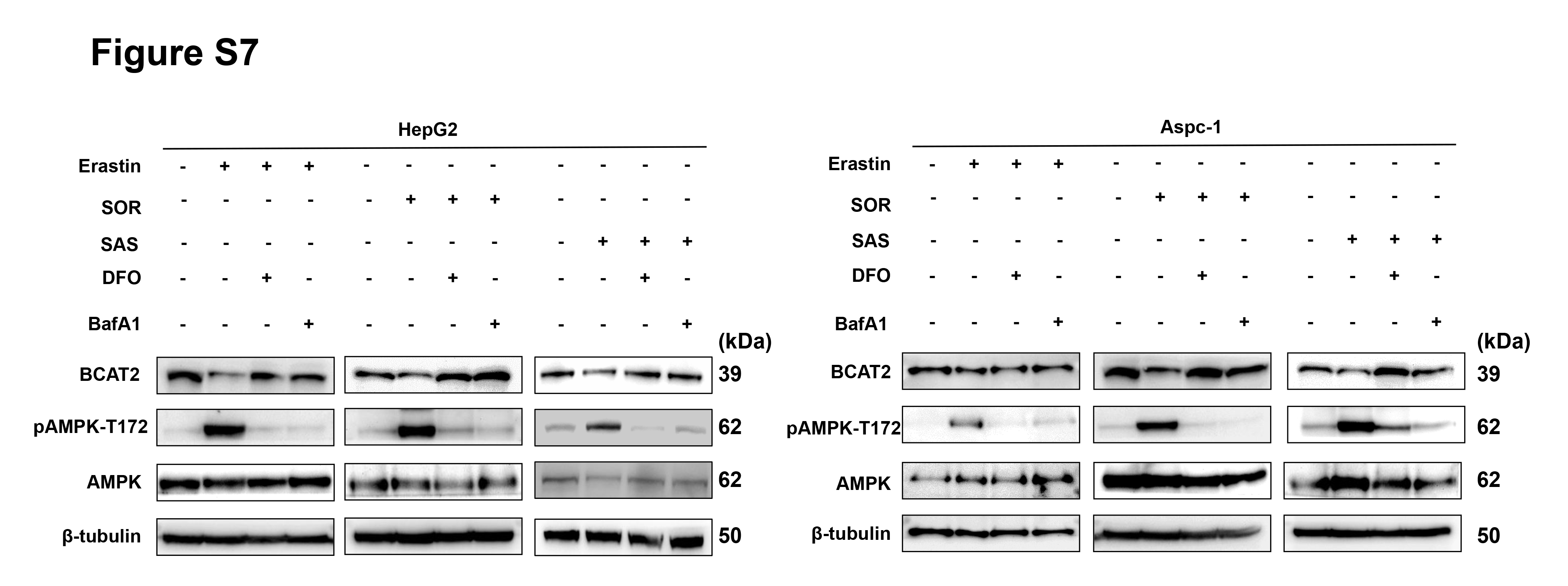

Supplement: Supplementary file 8 — Figure S7 [file 41418_2020_644_MOESM8_ESM.tif]

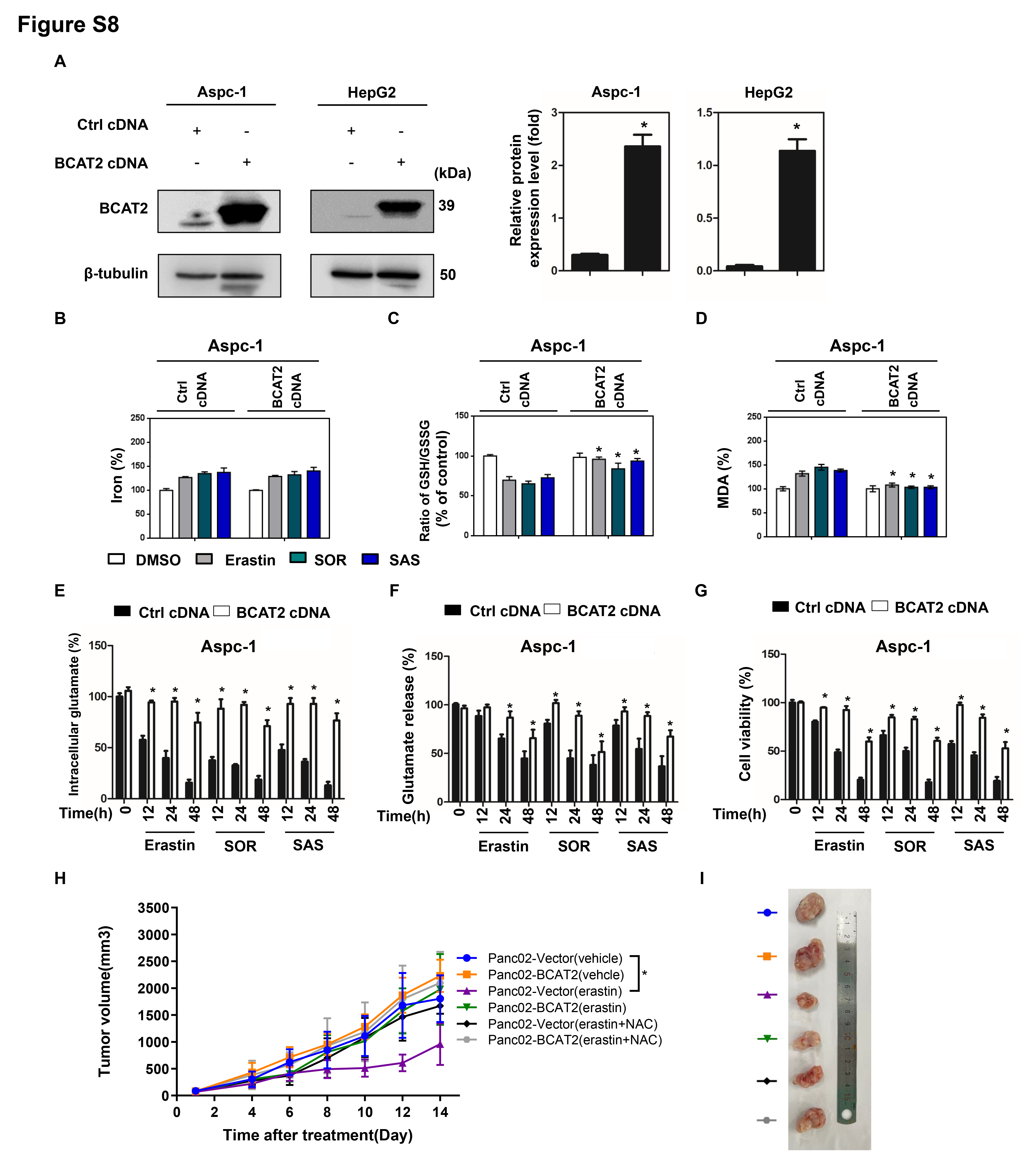

Supplement: Supplementary file 9 — Figure S8 [file 41418_2020_644_MOESM9_ESM.tif]

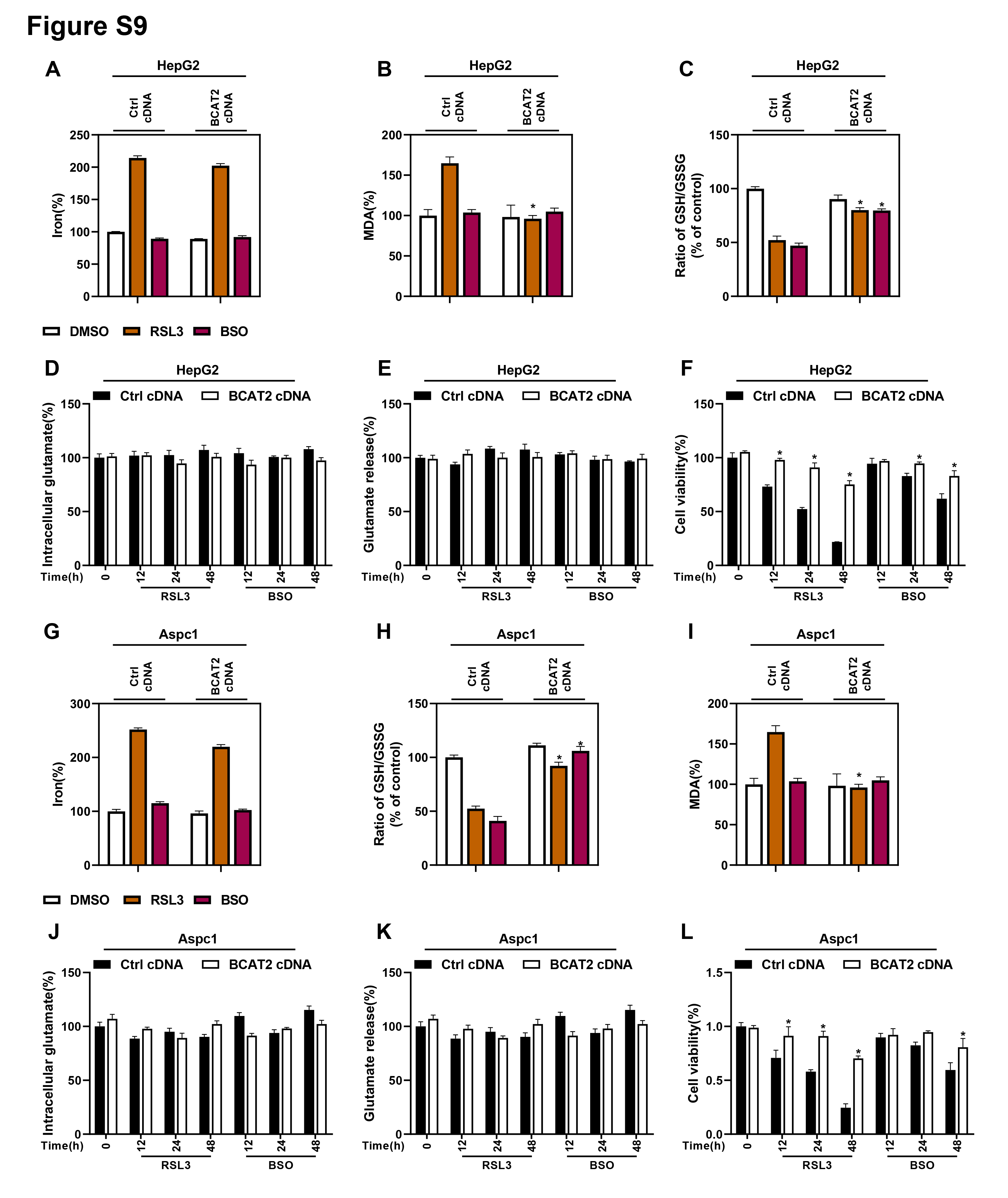

Supplement: Supplementary file 10 — Figure S9 [file 41418_2020_644_MOESM10_ESM.tif]

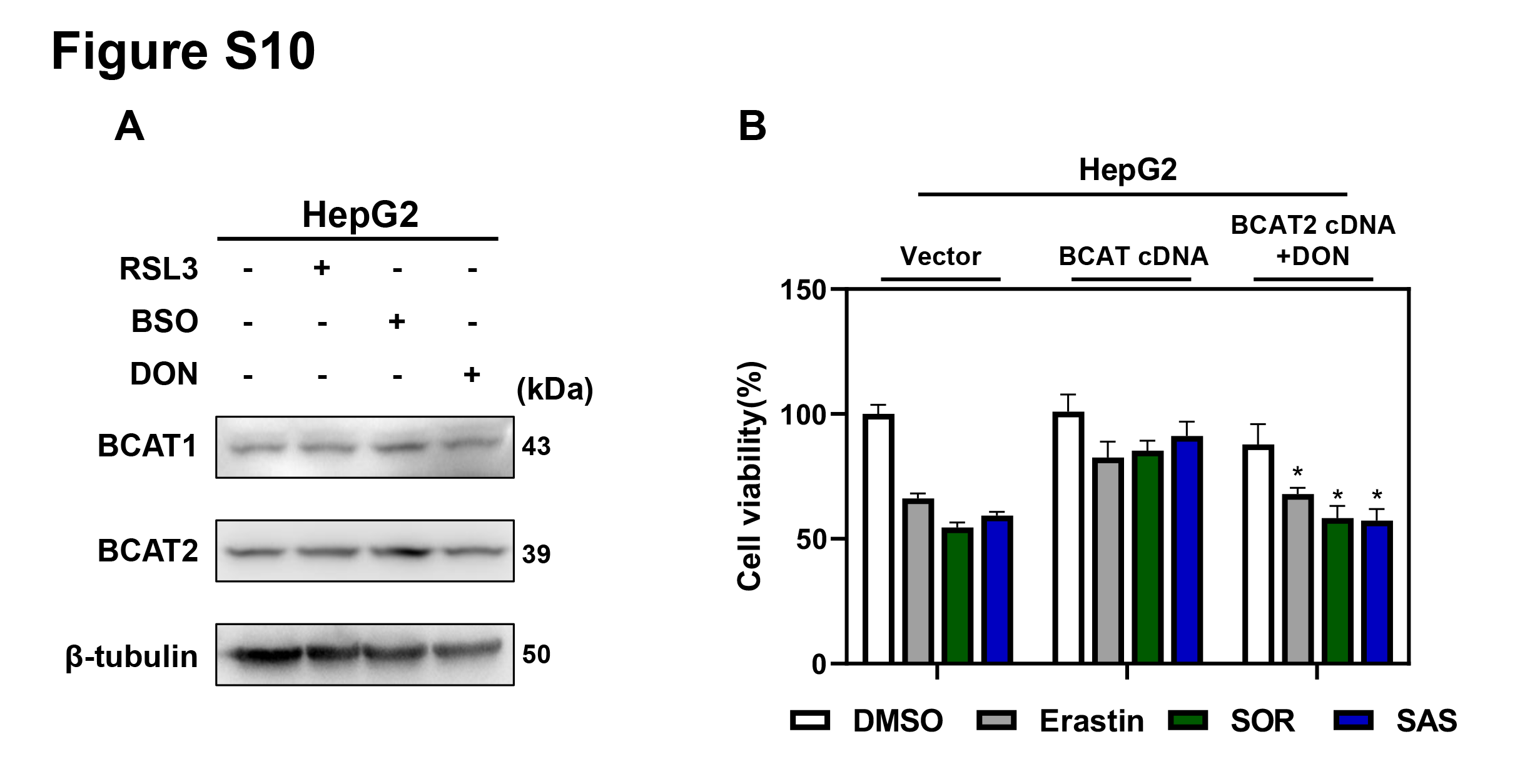

Supplement: Supplementary file 11 — Figure S10 [file 41418_2020_644_MOESM11_ESM.tif]

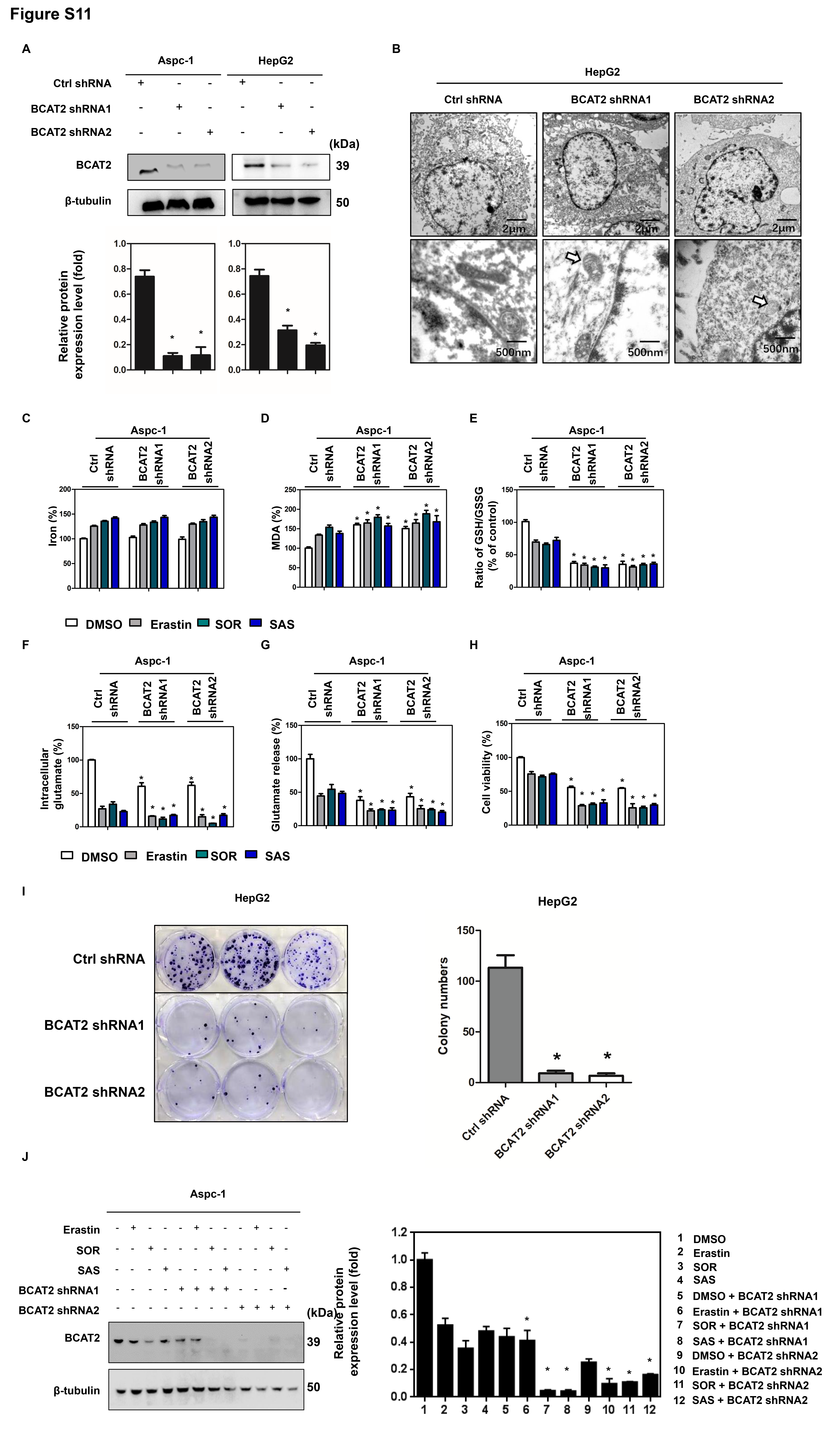

Supplement: Supplementary file 12 — Figure S11 [file 41418_2020_644_MOESM12_ESM.tif]

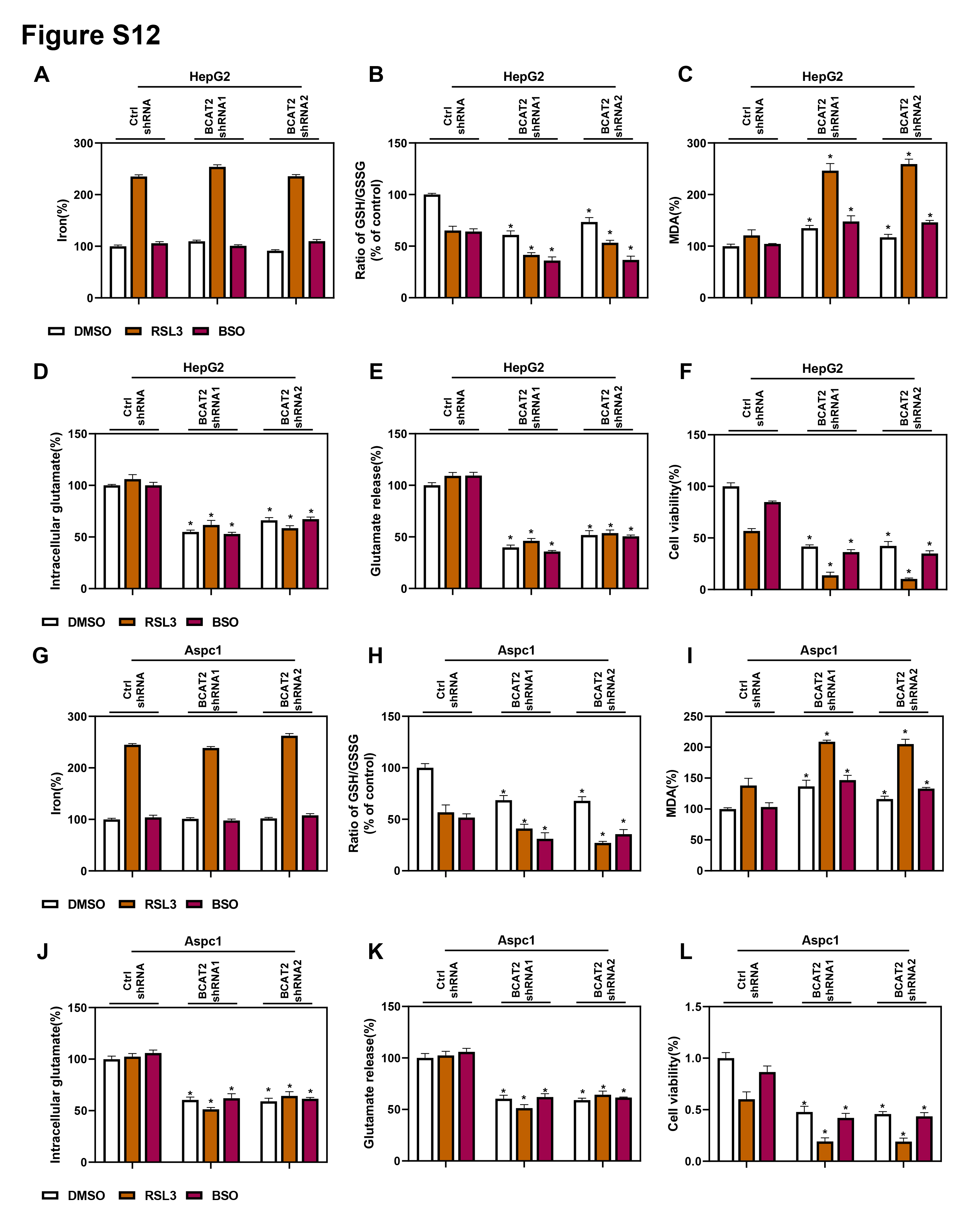

Supplement: Supplementary file 13 — Figure S12 [file 41418_2020_644_MOESM13_ESM.tif]

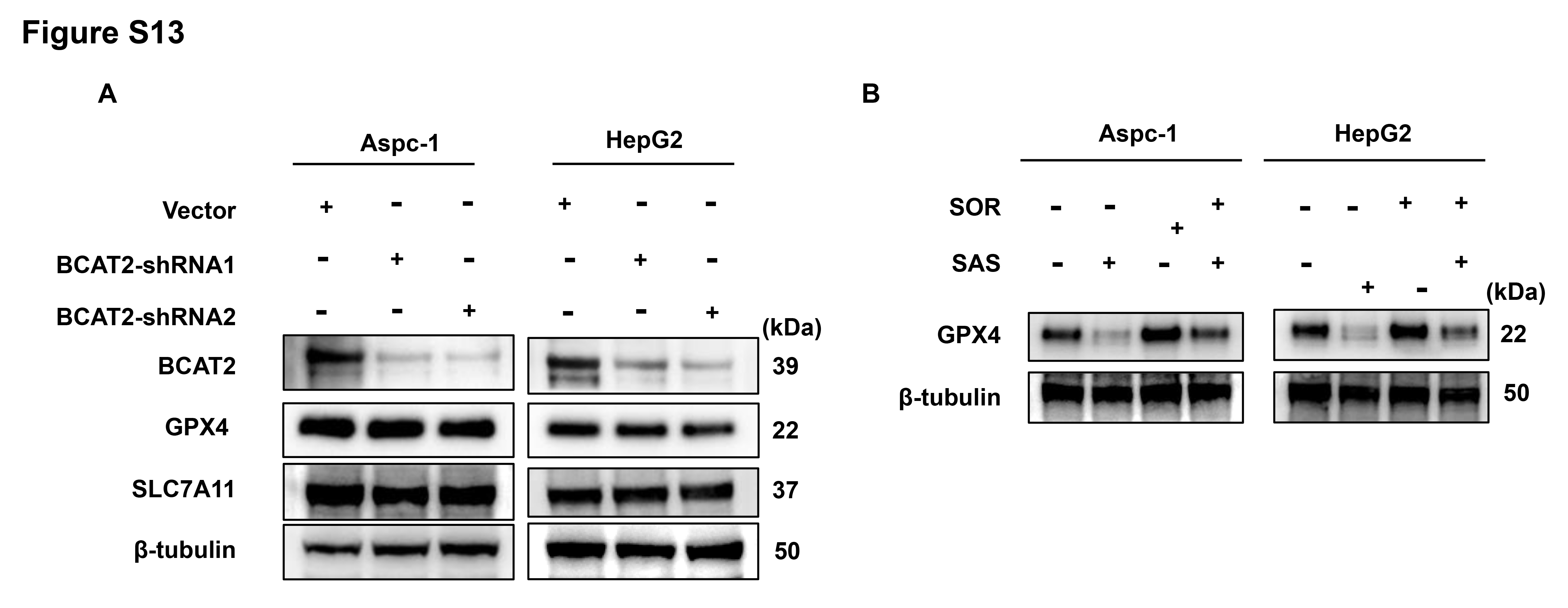

Supplement: Supplementary file 14 — Figure S13 [file 41418_2020_644_MOESM14_ESM.tif]

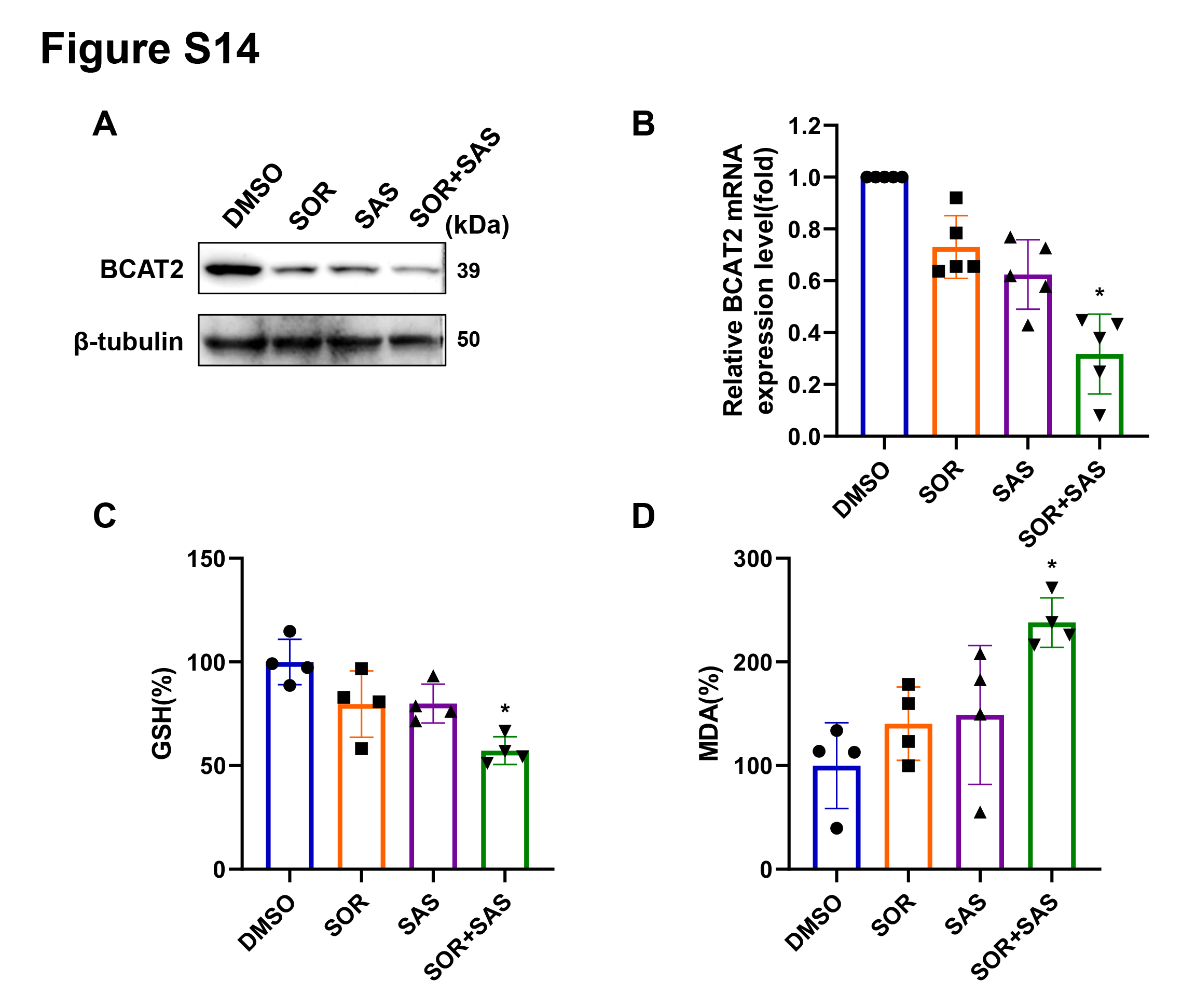

Supplement: Supplementary file 15 — Figure S14 [file 41418_2020_644_MOESM15_ESM.tif]
